# Decomposition of skin conductance data by means of nonnegative deconvolution

**DOI:** 10.1111/j.1469-8986.2009.00972.x

**Published:** 2010-07

**Authors:** Mathias Benedek, Christian Kaernbach

**Affiliations:** a Institut für Psychologie, Christian-Albrechts-Universität zu KielKiel, Germany; b Institut für Psychologie, Karl-Franzens-Universität GrazGraz, Austria

**Keywords:** Skin conductance, SCR, Electrodermal activity, EDA, Deconvolution, Decomposition

## Abstract

Skin conductance (SC) data are usually characterized by a sequence of overlapping phasic skin conductance responses (SCRs) overlying a tonic component. The variability of SCR shapes hereby complicates the proper decomposition of SC data. A method is proposed for full decomposition of SC data into tonic and phasic components. A two-compartment diffusion model was found to adequately describe a standard SCR shape based on the process of sweat diffusion. Nonnegative deconvolution is used to decompose SC data into discrete compact responses and at the same time assess deviations from the standard SCR shape, which could be ascribed to the additional process of pore opening. Based on the result of single non-overlapped SCRs, response parameters can be estimated precisely as shown in a paradigm with varying inter-stimulus intervals.

Sudomotor activity plays a major role in thermoregulation (Wenger, 2003) and in keeping the skin flexible for sensory discrimination (Jänig, 2006), but it is also a known concomitant of emotional states such as arousal (Boucsein, 1992). Sweat secretion alters the electrical properties of the skin, which is referred to as electrodermal activity (EDA). By applying constant voltage, the change in skin conductance (SC) can be measured non-invasively (Fowles et al. 1981). In psychophysiological research, EDA is one of the most commonly used response systems (Dawson, Schell, & Filion, 2007). EDA measures are applied in a wide range of issues in basic research (e.g., attention, emotion) as well as in clinical research (e.g., schizophrenia).

## Physiology of Sweat Secretion

Central control of sweat gland activity is attributed to the hypothalamic areas, especially the paraventricular and posterior nuclei (Boucsein, 1992). However, many other subcortical and also cortical regions are known to be involved in the modulation of its activation (Critchley, Elliott, Mathias, & Dolan, 2000; Edelberg, 1973;). Sudomotor fibers descend via hypothalamic-reticular-spinal sympathetic pathways in close proximity to other sympathetic fibers (e.g., vasoconstrictor or piloerector efferences) ending at the preganglionic sudomotor neurons. The preganglionic neurons leave the spinal cord ipsilaterally via the lateral horn leading to the sympathetic trunk, where they are switched to postganglionic neurons. The postganglionic neurons innervate the secretory part of the sweat glands in a widely ramified way. Though postganglionic sympathetic transmission is usually adrenergic, postganglionic sudomotor transmission is cholinergic, using acetylcholine as a synaptic transmitter. The secretory segment of the sweat gland is located in the subcutis. The sweat is discharged into a sweat duct leading through dermis and epidermis and ending in a pore on the skin surface. The density of eccrine sweat glands varies markedly over the human skin. Following Millington and Wilkinson (1983) the density is highest on the sole and dorsum of the foot, on the forehead, cheek, palm, and forearm (200–600 per cm^2^).

Postganglionic sudomotor fibers are slow unmyelinated fibers with conduction velocities of about 0.5 to 2 m/s (Decomyn, 1998; Schmelz, Schmidt, Bickel, Torebjörk, & Handwerker, 1998;). Conduction time from central activation to the sweat glands of the fingertips (with a mean distance of 1.1 m) was estimated at 1.1 s (Lim, Seto-Poon, Clouston, & Morris, 2003). Neuroeffector time at the sweat glands was estimated to take 348 ms (Kunimoto, Kirnö, Elam, & Wallin, 1991).

Each sweat gland is innervated by many different fibers (Kennedy, Wendelschafer-Crabb, & Brelje, 1994; Riedl, Nischik, Birklein, Neundörfer, & Handwerker, 1998;), and vice versa each sudomotor unit innervates a skin area of about 1.28 cm^2^ (Schmelz et al., 1998). Sweat glands differ markedly in their activity and seem to possess different activation thresholds (Nishiyama, Sugenoya, Matsumoto, Iwase, & Mano, 2001). Studies involving microneurography (Vallbo, Hagbarth, & Wallin, 2004) show that postganglionic sudomotor fibers fire in a burst fashion with bursts having a mean duration of 638 ms (Macefield & Wallin, 1996). A sudomotor burst corresponds to a single skin conductance response (SCR). The amplitude of the SCR was found to be linearly related to the amplitude of the integrated sudomotor nerve activity reflecting the frequency of action potentials (Bini, Hagebarth, Mynninen, & Wallin, 1980; Sugenoya, Iwase, Mano, & Ogawa, 1990;). Moreover, the SCR amplitude was shown to be related to the number of recruited sweat glands (Freedman et al., 1994; Nishiyama et al., 2001;). Sudomotor activity is known to be modulated by respiration and the cardiac cycle (Macefield & Wallin, 1996, 1999).

## Quantification Methods

The skin conductance signal is usually described as consisting of a slowly varying SC level (SCL), which is superposed by separate phasic SCRs. One is commonly interested to measure the amplitude of the phasic response to a given stimulus. Conventionally, SCRs that arise within a predefined response window (1–3 s to 1–5 s after stimulus onset) and that also meet a minimum amplitude criterion (0.01 to 0.05 μS) are considered to be elicited by the stimulus (Dawson et al., 2007; Levinson & Edelberg, 1985;). Estimation of SCR onset and amplitude is usually based on trough-to-peak analysis. Minima and maxima in the SC data are viewed as onset and peak latencies, and the amplitude is computed as the difference of the SC values for onset (trough) and peak times (Boucsein, 1992; Edelberg, 1967;). Some investigators are also interested in measuring temporal characteristics of the SCR. Parameters of foremost interest involve SCR latency (i.e., time from stimulus onset to SCR onset), rise time (time from SCR onset to SCR peak), and half recovery time (time from SCR peak to 50% recovery of SCR amplitude). Finally, parameters of tonic activity can be computed. The SCL can be estimated for adequate time intervals by averaging SC scores in segments free of SCRs (Boucsein, 1992). As an alternative, the frequency of nonspecific SCRs can also be assumed to reflect tonic electrodermal activity (Venables & Christie, 1980).

The scoring of phasic parameters is complicated for SCRs that occur in close temporal proximity, since succeeding SCRs will be distorted by the recovery slope of preceding SCRs. The degree of distortion depends on the amplitude and proximity of the preceding SCR (Grings & Schell, 1969). If the recovery slope cannot be extended and subtracted, the peak latency and the amplitude of the following SCR will generally be underestimated. Moreover, close superposition of two SCRs can obscure the onset of the latter or even make them appear as one. This is especially problematic if, as a consequence, SCRs are wrongly shifted outside or inside the response window and thus become misclassified responses as a whole. Paradigms with short inter-stimulus intervals (ISI) appear particularly prone to these errors. So far, different efforts have been made to identify superposing SCRs (Foerster, 1984; Thom, 1988;). Moreover, first approaches for the decomposition of SC data have been proposed. Barry, Feldman, Gordon, Cocker and Rennie (1993) suggested linearly extending the baseline to a point below the following SCR peak. Recently, more sophisticated techniques have been presented trying to decompose SC data into discrete phasic components. Lim et al. (1997) proposed a curve-fitting method for the decomposition of 10 s segments of SC data. A four- to eight-parameter model is employed to fit tonic level, slope of preceding SCR, and shape of one SCR or two overlapping SCRs (more details can be found in the section on the SCR shape). The decomposition process requires visual inspection to determine in advance if the segment includes a residual slope and overlapping SCRs before a standard least-squares routine fits the model to the data. SCR measures can then be derived from the model parameters. Lim et al. (1997) report significant increases of amplitude (by 15%) and latency (by 140 ms) as compared to standard trough-to-peak method, and demonstrated the applicability of the method to settings with short ISIs (Lim et al., 1999).

Alexander et al. (2005) proposed an automated analysis method based on the mathematical process of deconvolution. They argue that SC data are the result of a convolution process of the activity of sudomotor nerves (corresponding to a driver function) and an impulse response shaped like a biexponential function. Deconvolving SC data by the response function reveals the driver function, which conforms to a sequence of discrete bursts having a much shorter time constant than the SCRs. Peak detection is performed on the driver function and time segments of separate peaks are extracted. Finally, isolated SCRs can be obtained by calculating the difference of the original SC data with a reconstructed signal, for which the respective segment is set to baseline. Phasic parameters (e.g., SCR amplitude) can then be computed from each single, non-overlapped SCR.

## The Shape of the SCR

When measuring SC by means of exosomatic DC-recording, an isolated SCR shows a monophasic course, which is characterized by a steep increase of SC and a slow recovery (Boucsein, 1992). There have been different attempts to quantitatively or qualitatively describe the course of the SCR. Quantitative approaches look for mathematical functions describing the course of the SCR over time. Lim et al. (1997) proposed a sigmoid-exponential four-parameter SCR model, which is defined as follows: 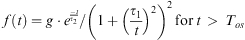
(1)where *g* reflects gain, τ_1_ and τ_2_ are related to rise time and decay time, respectively, and *T_OS_* is onset time. The sigmoid part serves as approximation of the cumulative action of sweat duct filling, while the exponential decay function is thought to depict the recovery limb. For each parameter, some rationale is provided that links it to physiological processes.

In the model of Alexander et al. (2005), the SCR is described by a biexponential function, which was previously used to model effects of individual nerve impulses on the synaptic activation of the neuronal membrane (Wright et al., 2001): 
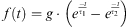
(2)

According to the authors, setting the parameter values τ_1_=0.75 and τ_1_=2 worked well for all data they analyzed. The model was found to adequately relate a short time-constant process (i.e., sudomotor bursts) to a long time-constant process (i.e., SCRs).

**Figure 1:**
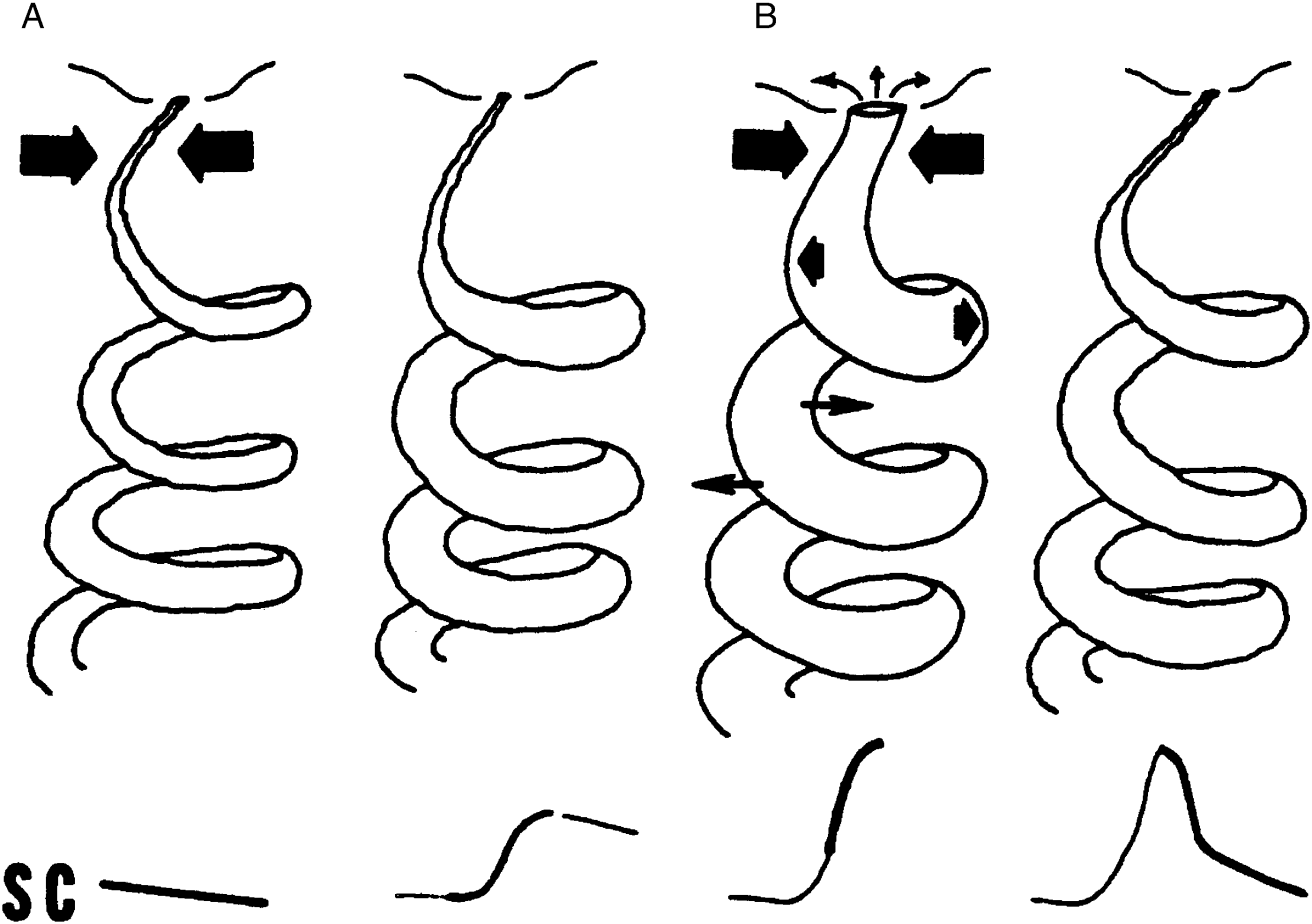
Diagram of two different sequences underlying an SCR (adapted from Edelberg, 1993, with friendly permission). If sweat ducts are filled to their limits, intraductal pressure will cause a hydraulic driven diffusion of sweat to the corneum resulting in a flat SCR (A). If intraductal pressure exceeds tissue pressure, the distal part of the duct and the pore will eventually open, which results in a peaked SCR (B).

A qualitative approach was taken in the *poral valve model* by Edelberg (1993). This model assumes an initial situation, in which especially the distal part of some or most of the sweat ducts are collapsed by the external pressure exerted by a well hydrated surrounding corneum and, consequently, most of the pores are closed. As sweat fills the ducts to the limits of their capacity, intraductal pressure will cause hydraulic driven diffusion of sweat into the corneum (see Figure 1A). Increasing hydration of deeper levels of the corneum will contribute to a moderately rising SC. As sweat is reabsorbed into the dermis or diffuses away from the periductal area, SC will slowly recover, resulting in a rather flat SCR. If secretion is strong enough and intraductal pressure becomes stronger than the tissue pressure of the corneum, the pore will eventually open. In addition to the diffusion into the corneum, sweat will now be forced out through the pore. Consequently, SC will show a steep increase. Volume loss through the pore is substantial, and, after a short time, the secretory rate cannot keep up with the loss of volume. The intraductal pressure will soon fall below tissue pressure, and pores will collapse again. This causes a rapid fall in SC, which finally passes into a slower recovery as in the abovementioned case (see Figure 1B). This model proposes that the SCR shape can primarily be ascribed to two different underlying processes: an unconditional diffusion process, which, taken alone, causes a rather flat SCR, and an optional opening of pores, which will add a steep peak to the basic SCR shape. These processes thus give rise to a variability of the SCR shape, which has not yet been properly accounted for in quantitative models.

## Modeling the Diffusion Process

The quantitative approaches described above (Equations 1 and 2) have been proposed based on the similarity of the resulting functions with experimentally obtained SCRs. Biexponential functions (Equation 2) can, however, be derived directly from models of the dynamics of the concentration of sweat in the corneum. It can be assumed that this concentration is governed by the laws of diffusion (Edelberg, 1993; Schneider, 1987;). Its dynamic can be modeled by a two-compartment model. The substance (sweat) is released to compartment A (sweat duct), transgresses to compartment B (corneum), and is eliminated from compartment B (e.g., by evaporation). Both diffusion and elimination are assumed to operate one way at speeds proportional to the concentration in their respective compartment. One-way diffusion can reasonably be assumed, if compartment B is much larger than compartment A. The dynamics can then be derived from two coupled first-order differential equations describing the concentration in compartments A and B: 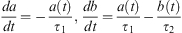
(3)

The assumption of forward-only diffusion allows a stepwise solution, entering the solution to the previous compartment in the equation for the next compartment. The solution of this coupled first-order equation is a biexponential function *b(t)*, the so-called Bateman function: 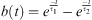
(4)

The Bateman function is characterized by a steep onset and a slow recovery. The steepness of onset and recovery is characterized by the time constants τ_1_ and τ_2_. This function is well known in pharmacokinetics to quantify the time course of first-order invasion of a drug into, and its first-order elimination out of, a compartment body model (Garret, 1994).

As mentioned before, a biexponential function was previously proposed by Alexander et al. (2005) as a model for the SCR shape (compare Equation 2). We prefer it to the exponentially damped sigmoid proposed by Lim et al. (1997), because its exact shape can be derived from a simple diffusion model. With respect to Edelberg's model, we assume that the Bateman function primarily describes the diffusion part of the SCR shape. Up until this time, we do not have clear assumptions that enable us to additionally model the effect of pore opening in a quantitative way.

We agree with Alexander et al. that deconvolution represents a very useful technique to reveal the discrete activation underlying SC data. However, standard deconvolution requires the assumption of a standard SCR shape (i.e., impulse response) and thus cannot account for different SCR shapes as they may result from the conditional process of pore opening. Therefore, we propose a deconvolution method that deconvolves data by a fixed response function (i.e., the Bateman function), but which at the same time allows the detection of any deviation from this SCR shape.

## Nonnegative Deconvolution

Standard (one-dimensional deterministic) deconvolution can be applied to any signal, which evolved from convolution of a driver function with an impulse response. If the shape of the impulse response is known, deconvolution will perfectly retrieve the original driver function. In accordance with Alexander et al. (2005), we assume that SC can be viewed as the result of a driver function (i.e., the activity of sudomotor neurons or sweat glands) triggering an impulse response (i.e., increased conductivity of the skin due to perfusion by sweat). Sudomotor neurons are supposed to be either active (stimulating sweat glands to discharge sweat) or to be inactive. From this, it can be postulated that a driver function, representing the activity of sudomotor neurons, should be nonnegative, either showing positive deflections (impulses) in states of activity or remaining at zero otherwise. Moreover, we assume that single SCRs correspond to discrete bursts of sudomotor activity and that consequently impulses should be compact in time. They shall thus exhibit a marked onset and offset which define their extent in time. These claims result in a nonnegative driver function, which is characterized by a zero baseline intermitted by discrete positive impulses with a compact support.

**Figure 2:**
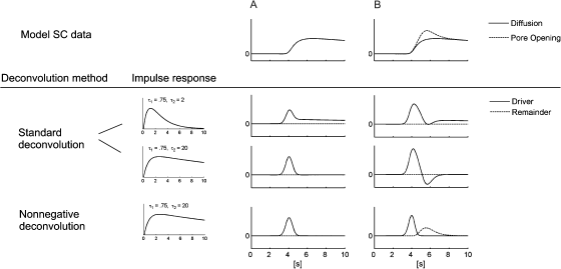
Standard deconvolution and nonnegative deconvolution applied to model data of two differently shaped SCRs: (A) flat SCR, resulting from sweat diffusion only; (B) peaked SCR, resulting from sweat diffusion and additional pore opening. Standard deconvolution is depicted for two different impulse response functions (Bateman function with small and higher τ_2_), while nonnegative deconvolution is only depicted for the latter impulse response function.

Systematic analyses of SC data by means of standard deconvolution employing the Bateman function for varying parameters (τ_1_, τ_2_), however, fail to produce such a driver function. This may be attributed to the fact that standard deconvolution only accounts for a fixed impulse response (i.e., a single standard SCR shape) and thus is not able to deal with varying SCR shapes. Typical deconvolution results for standard deconvolution applied to either flat or peaked SCR shapes (which may result from closed or open pores, as suggested by Edelberg, 1993) are displayed in Figure 2. Using an impulse response with small τ_2_ (e.g., τ_2_=2, as proposed by Alexander et al., 2005) yields a nonnegative driver function for flat and peaked SCR shapes; however, impulses show a tail after the main deflection, thus violating the assumption of compact impulses. On the other hand, using an impulse response with higher τ_2_ (e.g., τ_2_=20) will yield a compact impulse for a flat SCR shape; however, for a peaked SCR shape, the impulse shows a negative bend after the main deflection, thus violating the assumption of nonnegativity.

In order to comply with the claims of nonnegativity and compactness of impulses in the face of varying SCR shapes, we suggest a variant of the standard deconvolution method. Standard deconvolution can be thought to work in a similar way to arithmetic long division (Evans, 1995). The signal is virtually divided by the impulse response yielding the driver function. However, the procedure only accounts for the respective first digit of dividend and divisor and, since real numbers are allowed, there is no carryover. Therefore, negative values can occur in the quotient (i.e., driver function) and the remainder is zero. If we demand that all values of the quotient have to be nonnegative, we have to consider the entire divisor, calculate the quotient digit by digit, and take the overall minimum. This adapted version of deconvolution is even more similar to arithmetic long division since it considers the whole divisor. As there is still no carryover, the remainder is no longer confined to zero. Since this method ensures that the resulting driver is nonnegative, we may refer to it as *nonnegative deconvolution*. An explicit demonstration of the computation procedure for standard and nonnegative deconvolution, based on an example, is provided in the Appendix.

In the bottom row of Figure 2, nonnegative deconvolution is applied to flat and peaked SCRs using an impulse response with high τ_2_. Standard and nonnegative deconvolution do not differ for a flat SCR. For a peaked SCR, nonnegative deconvolution yields a nonnegative driver function exhibiting a compact positive impulse, plus a positive remainder. It thus, again, meets the claim of nonnegativity and compactness. The optional positive remainder represents a deviation from the basic SCR shape. Following Edelberg's model, a deviation from the basic (i.e., flat) SCR shape may be associated with the process of pore opening. According to this reasoning, the method would be able to segregate a peaked SCR into a diffusion part and an additional part attributed to the process of pore opening. Note that deconvolution by a response function with a total integrated area of 1 is a neutral transformation with respect to the area of the signals. Thus, the area of the phasic data equals the area of the sum of driver and remainder function; and the area of single SCRs equals the total area of the corresponding impulse and the corresponding remainder component. However, the signal-to-noise ratio is enhanced in the deconvolved signal as the time basis for single components is markedly reduced and the peak amplitude is enlarged.

## Method

### Participants

Forty-eight healthy student volunteers (29 females) were recruited from the Christian-Albrecht University student body through advertisement. Mean age of the participants was 22.8 years (*SD*=2.20). Prior to the experiment, the participants were presented single noise bursts of 85, 90, and 95 dB SPL (white noise of 140 ms total duration with 20 ms linear ramps). They were told that the experiment comprised thirteen noise bursts of 95 dB. After this instruction, participants could decide upon participation. No participant refused to participate. All participants gave their written informed consent and were paid for their participation. The procedure was approved by the ethics committee of the German Psychological Society.

### Equipment and Data Acquisition

The experiment took place in a soundproof cabin. The stimuli were presented via a closed Beyerdynamic DT 770 PRO headphone (Heilbronn, Germany). A 16-channel bioamplifier (Nexus-16; Mind Media B.V.; Roermond-Herten, The Netherlands) providing 24 Bit A/D conversion was used for data acquisition. A customer-specific SC sensor was used for SC recording, ensuring the acquisition of completely raw, unfiltered SC data. The functional circuit of the sensor comprised a voltage source of 10 V connected in series with 13.2 MΩ. Thus, for SC in a typical range of 1 μS or higher the sensor maintained a voltage of less than 0.8 V between the two flat Ag-AgCl electrodes of 10 mm diameter placed at the medial phalanges of the digits III and IV of the non-dominant hand. In order to preserve the natural condition of the skin, no isotonic electrode paste was used in this experiment. SC data was sampled at 32 Hz. As part of a standard routine, blood volume pulse was recorded via a photoplethysmograph placed on digit II of the non-dominant hand (sampled at 128 Hz), and respiration was assessed via a respiration belt placed on the chest (sampled at 32 Hz). The data of these additional sensors, however, were not analyzed.

### Experimental Task and Procedure

After a rest period of 3 min, the participants were presented with a series of thirteen noise bursts. The level of these noise bursts was 95 dB SPL, the total duration was 140 ms with 20 ms linear ramps. The sequence started with an initial stimulus, which was succeeded by stimuli that were virtually grouped into three blocks of four bursts. In each block, an ISI of 4, 8, 16, and 32 seconds was realized exactly once. The ISIs within each block were randomized with the only condition that the first ISI of each block was not the same as the last of the preceding block, thus ensuring that two consecutive ISIs were different from each other for the whole sequence. The analysis section considers activity related to the final twelve stimuli, ignoring the initial stimulus.

Participants were seated in a chair with a neck-rest, with their non-dominant forearm placed on a soft armrest. After attachment of the physiological sensors, the participants were asked to find a comfortable position and to avoid any unnecessary movement during the experiment. During the experimental session, the experimenter sat outside of the cabin and monitored the stimulus presentation and the recorded physiological data. The experiment took about 40 min in total.

### Statistical Analysis

The minimum-amplitude criterion for inclusion of SCRs was set to 0.01 μS. SCR components that assumably correspond to pore opening will be referred to as *PO components* hereafter. PO components of amplitudes equal or higher than 0.005 μS are considered to be significant and included in the further analysis. In order to account for the positively skewed distributions of SCR amplitudes, the data were standardized with the formula SC^*^=log(1+SC) (Venables & Christie, 1980). The respective units are labeled as log μS. For ANOVA analyses, degrees of freedom were corrected by means of the Greenhouse-Geisser method where appropriate and Bonferroni post-tests were used for pair-wise comparison of means.

Seven participants (14.6%) failed to show significant SCRs to at least 50% of the stimuli, which may constitute a habituation effect caused by the frequent repetition of identical stimuli. In order to allow for a powerful statistical analysis, participants displaying this comparatively low response rate were excluded from the further analysis. The final sample thus comprised 41 participants.

### Decomposition Procedure

**Figure 3:**
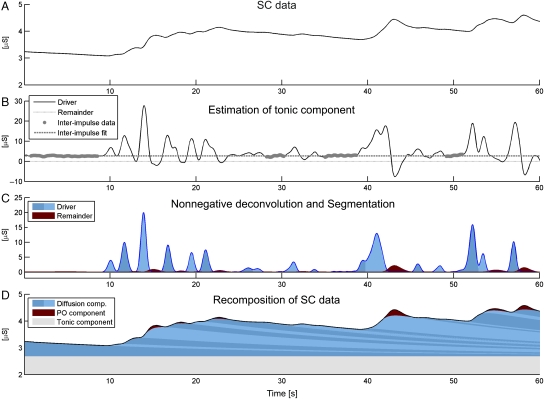
Sequence of the decomposition of SC data by means of nonnegative deconvolution. Given is a 60-s segment of raw SC data (A). Tonic SC activity is estimated based on inter-impulse data detected in the standard deconvolution of the raw SC data (B). Nonnegative deconvolution is applied to the phasic SC data (original SC data minus tonic SC activity) and single impulses and corresponding pore opening components are identified by means of segmentation of driver and remainder signal (C). The original SC data can finally be recomposed by superposition of its tonic and phasic components (D).

The decomposition procedure involves four steps: estimation of the tonic component, nonnegative deconvolution of phasic SC data, segmentation of driver and remainder, and reconstruction of SC data. The procedure is initially performed for a predefined parameter set (e.g., τ_1_=0.75, τ_2_=20). In order to increase the goodness of fit of the model, these parameters then become optimized, which involves rerunning all four steps for each new parameter set. In the following, the single steps of the decomposition procedure are described in more detail and exemplified on basis of a 60-s sample of SC data (see Figure 3). The entire decomposition is performed using our own SC analysis software (Ledalab 3.0.4) written in MATLAB, which is available online (http://www.ledalab.de).

#### Estimation of tonic component

Tonic EDA can be observed in the absence of any phasic activity (Boucsein, 1992). To this end, SC data is deconvolved, and all intervals that are not part of impulses (reflecting phasic activity) are used as estimates for the tonic activity. First, a standard deconvolution using the Bateman function as impulse response is computed. The resulting estimation of the driver is not nonnegative (Figure 3B, see also third row of Figure 2) which is undesirable. It does, however, only serve to find SC data free from phasic activity.

As data convolution can be conceived as a smoothing operation, deconvolution has the reverse effect and amplifies error noise. Therefore, the resulting driver function is smoothed by convolution with a Gaussian window (σ=200 ms). Then, peak detection is performed on the smoothed driver function in order to identify impulses. This is achieved by finding zeros in the first time-derivative of the smoothed driver function. A significant peak is detected if a local maximum has a difference of δ≥0.2 μS to its preceding or following local minimum. An impulse section is defined by the local minima preceding and succeeding the significant peak in time. All time sections that are not part of detected impulses (inter-impulse sections) are considered to reflect non-overlapped tonic component. Finally, tonic activity is estimated for a time grid with 100-s spacing by averaging the driver function values of available inter-impulse sections within the range of half of the grid spacing before and after the grid points. A cubic spline fit is used to interpolate the tonic activity based on the grid data (see Figure 3B). Thus, the tonic component is a quasi-steady function described by five parameters (point in time and four cubic parameters) every 100 s of data. Once the tonic component is estimated, it is subtracted from the raw SC data. This yields phasic SC data, which consists of a mere superposition of phasic components (i.e., SCRs).

#### Nonnegative deconvolution

In order to avoid initial deconvolution artifacts, which arise if the data do not start at zero level, the data is prefixed with a smooth data fade-in. To this end, the phasic data are extended by adding the rising part of the impulse response, such that the last sample of the extension (i.e., maximum of impulse response) matches the first sample of the phasic data. Then, nonnegative deconvolution is applied to the extended phasic data as previously described. It results in a nonnegative driver function and a nonnegative remainder. Both signals are smoothed by convolution with a Gaussian window (σ=200 ms). After smoothing, the discreteness of the driver impulses becomes evident (see Figure 3C).

#### Segmentation of driver and remainder

**Figure 4:**
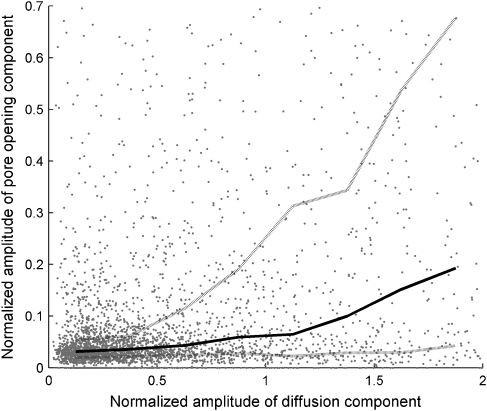
Normalized amplitudes of diffusion and pore opening components of SCRs, for all SCRs that occurred during the experiment. The solid and dashed lines show the median and the 25% and 75% percentiles for the size of the pore opening component for a given size of the diffusion component (in the range from 0 to 2 at bins of 0.25).

The obtained driver function is segmented in order to identify single impulses. This, again, is realized by a peak detection analysis as described earlier (now applying a less conservative detection threshold of δ≥0.01 μS). Remainder segments are allotted to a certain impulse if their onset falls into the time epoch from the onset of this impulse to the onset of the succeeding impulse. It can be observed that, if there is any deflection in the remainder segment, it reliably begins around the peak latency of the corresponding impulse and ends at the latest before the peak of the following impulse (see Figure 3C, compare also Figure 4B). This supports the concept of nonnegative deconvolution and its relation to the poral valve model. Thus, nonzero activity of the remainder function subsequent to an impulse could be attributed to the pore opening process of the respective response.

#### Reconstruction of SC data

The original SC data can now be recomposed from its components. Each SCR is reconstructed by convolution of the respective impulse with the impulse response function and by adding the respective PO component, if available. SCR characteristics (e.g., SCR amplitude) can then be derived from the shape of a single non-overlapped SCR. Finally, all SCRs are superposed to the tonic component, restoring the original SC data on the basis of a full decomposition (see Figure 3D).

#### Optimization

However, the most adequate realization of the response function (i.e., Bateman function) is unknown and thought to reflect individual differences in skin characteristics. Therefore, the initial parameter set is optimized to fit the observed SCR shape as accurately as possible. To this end, several criteria are used to evaluate the quality of the obtained model and to optimize it. First, the deflections of driver and remainder (i.e., impulses and PO components, respectively) and the signals should approach zero between deflections. As an indicator of discreteness (*d*), the number of succeeding samples with values above a predefined threshold (of δ=0.2 and 0.005 for driver and remainder function, respectively) are counted. These counts represent the length of a non-zero section. They are squared, summed, and divided by the total sample number. This function returns high values, if there are long time sections above threshold. Then, the number of phasic responses (*n*) is considered, as a higher number of responses allow for a closer fit, but also opposes the parsimony of the model. Finally, the *RMSE* of the difference between original SC data and recomposed data is used as an indicator of the goodness of the fit. Based on these criteria, a compound criterion *c* is computed as follows: 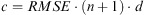
(5)

The model is optimized by minimizing the criterion *c*. Low criterion scores are obtained for a model based on a low number of short impulses offering a close fit to the original data. The optimization is achieved by means of a gradient descent method (e.g., Snyman, 2005), which essentially changes parameters in direction of highest criterion improvement until no further significant improvement can be obtained.

As exemplified in Figure 3, some SCRs do not show a distinct onset defined by a local minimum, but they correspond to a discrete impulse in the driver signal (e.g., the second or the one but last SCR). The decomposition method thus detects and segregates SCRs, which would have been missed or merged by standard peak detection.

## Results

### Decomposition of SC Data

Decomposition of SC data was automatically processed for all data. The reconstructed data fitted the original data with an average *RMSE* of 0.019 μS (*SD*=0.01). The optimization of τ parameters was performed for four different sets of initial values (τ=0.75, 2; τ=0.75, 20; τ=0.75, 40; τ=0.75, 60). All four sets usually converged toward a common solution, and the solution with the overall lowest model error was kept. The optimized values averaged for the sample were τ_1_=0.46, τ_2_=29.06 (*SD*=0.48 and 14.85, respectively).

Impulses could be discriminated at inter-impulse latencies (or inter-SCR latencies, respectively) down to 0.69 s. Overall, significant PO components were found for 44.1% of the above–threshold SCRs, and their amplitudes made up for 25.1% of the total SCR amplitudes.

Figure 4 shows the amplitudes of the PO components as a function of the amplitude of the underlying diffusion components. The amplitudes have been normalized by dividing them by the mean of the SCR amplitudes of the respective participant. Amplitudes of diffusion and PO components per participant showed an average correlation of *r*=.50 (*SD*=.25), suggesting a higher probability that large diffusion components are accompanied by large PO components. The median (solid line) and percentiles (dotted lines) also show that this positive relation is not linear over the total range. The probability for a PO component of a significant size increases disproportionately for diffusion components that have at least 50% of the mean amplitude for this participant. Moreover, the moderate and nonlinear correlation of diffusion and PO components are an indication of the high variability of the SCR shapes.

A recommended standard method to quantify the SCL is to average SC scores at the respective beginning of each SCR and average them over an interval of 10 s (Boucsein, 1992). This standard measure was compared to the tonic component (as obtained by the decomposition) with respect to the variability over time. The variability of the tonic activity was assessed by averaging the tonic activity for time intervals of 10 s and then computing the mean absolute difference of succeeding time intervals. The standard measure of SCL showed a mean variation of 0.14 μS (*SD*=0.08) per 10 s. The tonic component derived from the decomposition showed a markedly lower mean variation of only 0.03 μS (*SD*=0.02) per 10 s (*t*[40]=10.42, *p*<.001).

### Event-Related Activation

**Figure 5:**
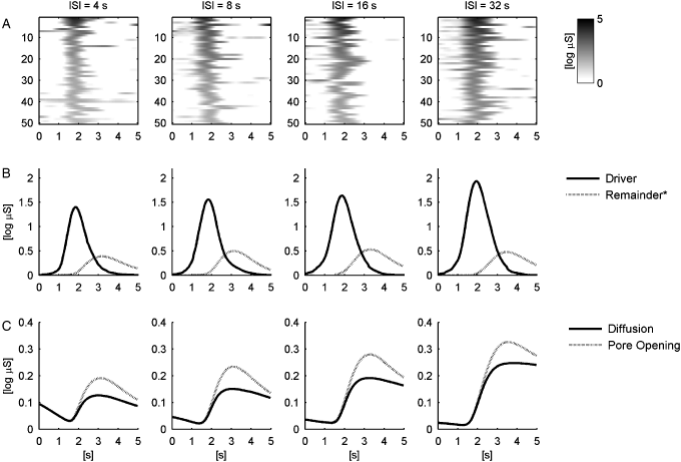
Event-related EDA in response to a startle probe for varying ISIs (4, 8, 16, and 32 s) depicted for 6 s relative to stimulus onset. Panel A shows the driver impulse for the 50 strongest responses. Panel B shows the average driver (impulse) and remainder data (*The remainder was amplified by factor 6 in order to be discriminable in a common scaling with the driver data). Panel C shows the SCR recomposed by diffusion and pore opening part.

Figure 5A shows prototypical driver impulses for the four different ISIs. With a few exceptions, most driver impulses have comparable onset times and durations. The width of the averaged driver impulses (Figure 5B) is thus mainly due to the width of the single pulses, and not to onset variability. Figure 5B shows averaged driver impulses and averaged remainder data for the four ISI conditions.

The event-related response was analyzed relative to stimulus onset. Response parameters were averaged per participant and analyzed with respect to the inter-stimulus interval (ISI=4, 8, 16, and 32 s) by means of ANOVA (within-subject factor ISI). Impulses occurred with a mean onset latency of 1249 ms (*SD*=246.4) and a mean peak latency of 2095 ms (*SD*=308.8). They showed a mean duration of 1615 ms (*SD*=416.8), a mean log-transformed amplitude of 1.04 log μS. While impulse onset and peak latencies were not influenced by ISI (*F*[3,117]=1.51, *p*=.22, ɛ=.88, η^2^=.04; and *F*[3, 117]=1.11, *p*=.35, ɛ=.76, η^2^=.03, respectively), impulses related to longer ISIs showed a longer duration (*F*[3,111]=4.17, *p*<.05, ɛ=.86, η^2^=.10) and a higher amplitude (*F*[3,117]=9.52, *p*<.001, ɛ=.79, η^2^=.20; see Table 1 and Figure 5).

**Table 1:** Means and Standard Deviations of Various Shape Characteristics of Impulse, PO Component and SCR by Inter-Stimulus Interval (ISI)

	ISI			
	4	8	16	32
	*M (SD)*	*M (SD)*	*M (SD)*	*M (SD)*
Impulse Onset				
latency (ms)	1311 (273)	1240 (283)	1225 (208)	1219 (219)
Peak latency (ms)	2057 (308)	2091 (344)	2106 (309)	2124 (272)
Duration (ms)	1458 (344)^a^	1610 (401)^ab^	1662 (474)^b^	1720 (419)^b^
Amplitude (log μS)	1.71 (0.73)^a^	1.89 (0.81)^ab^	2.04 (0.79)^bc^	2.21 (0.78)^c^
Pore opening				
Onset latency (ms)	1910 (368.1)	1848 (294.7)	1906 (331.7)	1973 (301.1)
Peak latency (ms)	3389 (526)^ab^	3302 (469)^a^	3442 (518)^b^	3648 (566)^bc^
Duration (ms)	4486 (986.1)	4257 (775.7)	4138 (850.4)	4302 (1121.1)
Amplitude (log μS)	0.11 (0.07)	0.13 (0.08)	0.13 (0.09)	0.12 (0.09)
SCR				
Amplitude (log μS)	0.22 (0.15)^a^	0.27 (0.18)^a,b^	0.31 (0.19)^b,c^	0.36 (0.20)^c^
Area (log μS·s)	1.84 (0.62)^a^	2.19 (0.61)^b^	2.24 (0.73)^b^	2.53 (0.71)^c^

*Note*^a,b,c^Means with different letters differ statistically according to Bonferroni post-tests.

The PO components occurred at a mean onset latency of 1938 ms (*SD*=282.6), which was on average 728 ms (*SD*=226.1) after impulse onset and 118 ms (*SD*=139.9) before impulse peak. Their mean peak latency was 3573 ms (*SD*=573.3), which is 709 ms after impulse offset. PO components showed a mean duration of 4296 ms (*SD*=1045.3), and their log-transformed amplitude amounted to 0.11 log μS (*SD*=0.74). Neither onset latency, duration, nor amplitude of PO components was found to differ with respect to ISI (*F*[3, 87]=1.42, *p*=.24, ɛ=.79, η^2^=.05; *F*[3, 84]=0.95, *p*=.42, ɛ=.78, η^2^=.03; *F*[3, 87]=0.85, *p*=.46, ɛ=.84, η^2^=.03). However, PO peak latency was found to be higher for increasing ISIs (*F*[3, 87]=8.78, *p*<.001, ɛ=.64, η^2^=.23; see Table 1 and Figure 5).

SCRs, which were reconstructed from impulses and PO components, have—per definition—the same onset latencies as the respective impulses. Their mean log-transformed amplitude was 0.28 log μS (*SD*=0.17) and their mean log-transformed area was 2.20 μS·s. SCR amplitude as well as SCR area were significantly higher for longer ISIs as compared to shorter ISIs (*F*[3,117]=13.51, *p*<.001, ɛ=.73, η^2^=.26; *F*[3,87]=11.11, *p*<.001, ɛ=.74, η^2^=.28; see Table 1 and Figure 5).

### Comparison of Nonnegative Deconvolution and Standard Peak Detection

The method of nonnegative deconvolution detected SCRs in 87% of the events and was found to be more sensitive than the standard peak detection method displaying a significantly lower detection rate of 78% (χ^2^[1]=113.8, *p*<.001). The difference in the detection rates was largest for the shortest ISI of 4 s (+11%) and lowest for the longest ISI of 32 s (+6%), the interaction of method and ISI was, however, not significant (*F*[3,120]=0.36, *ns*.).

For events at which both methods detected significant SCRs, nonnegative deconvolution yielded higher average SCR amplitudes (+0.06 log μS or +17%) and shorter SCR latencies (−339 ms or −21%) as compared to standard peak detection (*M*=0.30 log μS, *SD*=0.16 vs. *M*=0.26 log μS, *SD*=0.15, *t*[40]=11.31, *p*<.001, and *M*=1227 ms, *SD*=170 vs. *M*=1565 ms, *SD*=214, *t*[40]=−19.89, *p*<.001).

## Discussion

Skin conductance is a widely used measure of psychological arousal. Its popularity may partly be due to the availability of simple and inexpensive methods for data acquisition (Dawson et al., 2007). Moreover, phasic skin conductance responses seem to be a reliable concomitant of states of arousal, with SCR amplitudes providing information on the intensity of these states. While data acquisition can be achieved quite easily, data analysis is faced with a complex signal of many superposed discrete reactions overlying a tonic component. Pragmatic but imprecise methods, such as trough-to-peak analysis, are commonly applied to extract parameters from raw SC data.

Recently, there have been valuable efforts to decompose SC data into single components striving to avoid distortions of parameter estimations due to superposed SCRs. Lim et al. (1997) proposed a fitting method for decomposing 10-s sequences of data into a sloping baseline and up to two overlapping SCRs. Alexander et al. (2005) introduced the deconvolution method, which assumes a standard SCR shape and transforms SC data into a sequence of discrete impulses, which are then used to restore single non-overlapped SCRs.

The method proposed in this paper extends prior approaches and is aimed at decomposing comprehensive SC data sets (e.g., data of a complete experimental session) into a tonic component and a sequence of discrete phasic components allowing for a variable SCR shape. It is based on Edelberg's (1993) conception of two mechanisms contributing to the SCR shape, namely, sweat diffusion and pore opening (i.e., sweat expulsion). The process of sweat diffusion was modeled by a two-compartment diffusion process. It results in a biexponential function, which is assumed to capture the essential SCR shape based on sweat diffusion. Variations of this basic shape (due to pore opening) can be assessed by means of nonnegative deconvolution. Nonnegative deconvolution (as compared to standard deconvolution) yields a driver signal, which fulfills the constraints of nonnegativity. If used with the appropriate template, it results in compact impulses. It is thus in accordance with the assumption of a nonnegative sudomotor nerve activity characterized by discrete bursts. In addition, nonnegative deconvolution also yields a nonnegative remainder signal, which captures all variations from the basic SCR shape. Discrete peaks in the remainder signal in close proximity to peaks of the driver function are assumed to represent the physiological process of pore opening, which occasionally accompanies sweat diffusion in an SCR.

In the present experimental study, noise bursts (i.e., startle probes) were used to elicit well-defined SCRs at varying inter-stimulus intervals (ISIs) of 4 to 32 s. All data were successfully decomposed into a slowly varying tonic component (i.e., SCL) overlapped by a sequence of SCRs by means of automated analysis. Optimization of individual model fit resulted in average estimations for SCR shape parameters of τ_1_=0.46, τ_2_=29.06. A diffusion process described by these parameters shows a half-recovery time of 22.5 s. For such a slow recovery speed, it can easily be seen that a tight sequence of SCRs will rapidly accumulate and result in an overall increase of SC. On this note, it appears possible that the variation of the SCL—as assessed by standard techniques (e.g., SC value at SCR onset; see Boucsein, 1992)—is determined by the slow diffusion process of sweat through the corneum and thus largely represents a reverberation of phasic responses. This notion is corroborated by the observation that the variability of the decomposed tonic component was markedly reduced as compared to a standard measure of SCL. What still is captured by the decomposed tonic component may be attributed to some very slow physiological or non-physiological processes, which do not necessarily reflect SCL in the sense of tonic activation any more.

The characteristics of single SCRs were studied on the basis of well-defined responses to startle probes. The average impulse onset latency (i.e., SCR onset latency) was 1249 ms, which is markedly shorter than the onset latency of 1703 ms recently reported by Lim et al. (2003) for 100 dB startle probes. This difference may be the result of the particular conditions for onset determination based on deconvolved data. Impulses have a very advantageous signal-to-noise ratio as compared to raw SC data. They arise from a quasi constant baseline, have high amplitudes, short rise times (846 ms) and thus exhibit much steeper inclinations than SCRs. To some extent, the difference could also be attributed to the effect of data smoothing, which is indispensable in a deconvolution procedure and which will tend to advance the onset of any deflection. This effect should, however, not exceed the temporal parameters of the smoothing procedure (Gaussian window with σ=200 ms in our study). As a single impulse appears to have a virtually symmetrical shape (with its peak 846 ms after impulse onset and a duration of 1615 ms), the impulse peak latency is not prone to distortion by smoothing. Therefore, if we want to tell if an SCR is located within a predefined response window, the impulse peak latency may represent an even more reliable indicator of SCR position than impulse onset.

A driver impulse is assumed to be a correlate of the activity of the sudomotor nerves (Alexander et al., 2005). The mean impulse duration of 1615 ms, however, is markedly longer than the mean duration of heat-induced sudomotor bursts (638 ms) reported by Macefield and Wallin (1996) on grounds of microneurographical methods. Although one generally has to stay cautious when directly comparing data resulting from such different recording techniques as microneurography and SC recording, this discrepancy might also be attributed to the different stimulus type. Macefield and Wallin used an initial warming procedure in order to elicit spontaneous responses. These spontaneous responses might be weaker than SCRs elicited by startle probes. While smoothing might feign longer durations, this effect will again be limited to the width of the smoothing window. In order to avoid smoothing biases, the impulse duration could also be estimated by means of the second moment of the respective impulse section.

SCR amplitudes were found to increase with increasing ISI. Short ISIs will result in stronger superposition of SCRs, which may lead to a higher underestimation of SCR amplitudes. This is different for the decomposed signal. Even for an ISI as short as 4 s, the event-related driver impulse (in contrast to the SCR) does not show any overlap or distortion caused by the preceding response (see Figure 5). We can, therefore, assume that response parameters retrieved by the nonnegative deconvolution method are largely unaffected by responses dating back at least 4 s and were measured without significant distortion. As quantification bias can be ruled out, this effect has to be attributed to psychological or physiological reasons, such as increased excitability and reduced refractoriness (Recio, Schlacht, & Sommer, 2009). This provides an interesting opportunity to study which response characteristics covary with SCR amplitude. Higher SCR amplitude (as found for higher ISIs) goes along with increased duration and amplitude of impulses and with a deferred PO peak. The latter effect might be the result of increased impulse duration. As sweat secretion prevails for a longer time (which is indicated by increased impulse duration), the average peak of PO activity is expected to be postponed. Both increased impulse duration and impulse amplitude will lead to increased impulse area. Thus, the impulse area may be considered as a meaningful compound measure of impulse size. Moreover, impulse area plus area of corresponding PO components equal the total SCR area. An area measure was already suggested to represent a more comprehensive measure for the “strength of affect” (Traxel, 1957). However, maybe due to difficulties in the unambiguous computation of the SCR area, area measures have not yet been adopted by psychophysiological literature (Boucsein, 1992).

According to Edelberg's (1993) poral valve model, variations in the shape of the SCR can primarily be ascribed to the varying contribution of pore opening. SCRs with open pores will have a peaked shape, whereas the SCR shape will be rather flat when the pores stay closed. The results obtained with nonnegative deconvolution appear to be in line with this model. The onset of the average remainder deflection depicted in Figure 5B roughly coincides with the peak of the corresponding driver impulse, and the deflections are limited in their temporal extent. This is compatible with the notion that they reflect the effect of pore opening following secretion into the sweat duct. Pore opening should be more likely for large driver impulses, leading to the secretion of large quantities of sweat. This is reflected by the positive correlation between driver impulse amplitude and the existence and size of a PO component (see Figure 4). Interestingly, the relation of the amplitudes of diffusion and PO components does not appear to be linear over the total range. This might be due to the fact that the poral valve needs a certain minimum pressure to open. Moreover, the correlation between diffusion component and PO component is only moderate, with examples of high PO components following small diffusion components and vice versa. Nonnegative deconvolution thus reveals the high variability of SCR shapes and also provides means of dealing with it. A possible cause for this variability might be that pore opening does not only depend on the intensity of a single response, but also on the total discharge pattern including especially preceding responses that might have left the sweat ducts prefilled, and on the hydration of the skin that influences the elasticity and tension of the valve.

In conclusion, the poral valve model may serve to explain deviations from standard SCR shapes, which conform to the remainder of nonnegative deconvolution (i.e., PO components). This notion, however, has to remain somewhat speculative in the absence of direct empirical support. Direct observation of the sweat pores and concomitant sweat secretion will be necessary to decide upon the adequacy of this model and its applicability to the results obtained by nonnegative deconvolution. This could be achieved by combining standard SC measurements with videomicroscopy, a method which was already successfully implemented by Nishiyama et al. (2001).

A comparison of the proposed decomposition method to standard peak detection revealed that the former yields enhanced detection sensitivity for elicited SCRs, and the benefit was highest for the shortest ISI of 4 s. In the case that both methods detected SCRs, nonnegative deconvolution returned larger SCR amplitudes and reduced SCR latencies. This result pattern conforms to the notion that an SCR, which arises on the declining tail of its preceding SCR, will show reduced amplitude, postponed onset (to the point in time when the initial rise of an SCR surpasses the underlying decline), and it may even fail to be detected without the use of decomposition. The increase of amplitude by decomposition was in the range previously reported by Lim et al. (1997). In contrast to the present results and the abovementioned expectations, Lim et al. also reported an increase of SCR latency as compared to standard scoring method. Besides these general effects, it is expected that a valid decomposition should also enhance the correct classification of SCRs to stimuli and thus reduce the error variance of the estimated event-related response magnitude.

In summary, nonnegative deconvolution adds to the power of decomposition of skin conductance data and extends previous approaches in at least three significant respects. First, it provides a way to employ the efficient method of deconvolution proposed by Alexander et al. (2005) and at the same time to retain the consideration of variable SCR shapes (compare Lim et al., 1997), even without a new estimation of basic SCR parameters for each single SCR. Second, all aspects of the mathematical model were derived from and thus are based on a physiological rationale. Finally, the decomposition can be applied to full-length data, separating all phasic components and also considering tonic activity. It thus results in a complete decomposition model of the original SC data. However, a further validation of the method and the underlying assumptions is necessary and should involve the combination of standard SC recordings with microneurographical methods or videomicroscopy. This will not only enable an even more valid decomposition of SC data but also meet the strive for a full understanding of the complex data recorded from the skin.

## APPENDIX

A demonstration and comparison of standard and nonnegative deconvolution is provided on the grounds of long division. It is assumed that the signal *f* is the result of the driver function *g* convoluted with the impulse response *h* plus some remainder *r* such that

Let *f* be (1,0,4,1,0, …) and *h* be (2,1).

In standard deconvolution, *r(i)* is assumed to be zero. Employing long division, *g(i)* is simply determined by *f(i)*/*h(i)*, thus ignoring further digits of *h*, and *g(i)* can take on real negative values. Deconvolving *f* by standard deconvolution yields:

**Table:**

	*g(i)*=	.5,	−.25,	2.125,	−.5625,	…	
2,	1	1,	0,	4,	1,	0,	…
	1,	.5				
		−.5,	4			
		−.5,	−.25			
			4.25,	1		
			4.25,	2.125		
				−1.125,	0	
				−1.125,	−.5625	
					−.5625	
					:	
*r(i)=*	0,	0,	0,	0		

In nonnegative deconvolution, it is assumed that *r*(*i*)≥0. Employing long division, *g(i)* is determined as

with *j*=*i* to *i+k*−1 and *k* being the number of digits of *h*. This procedure ensures that *g*(i) is nonnegative. Deconvolving *f* by means of nonnegative deconvolution thus yields:

**Table:**

	*g(i)*=	0,	0,	1,	0,	…	
2,	1	1,	0,	4,	1,	0,	…
	0,	0,				
		0,	4,			
		0,	0			
			4,	1		
			2,	1		
				0,	0	
				0,	0	
					0	
					:	
*r(i)=*	1,	0,	2,	0		

Assuming that *r* only shows compact deflections from zero, which have a shorter time constant than *h*, this method appears to be equally robust as standard deconvolution to deviations from the correct impulse response function *h* and to error noise.
